# *N*-(3-Chloro­phen­yl)benzamide

**DOI:** 10.1107/S1600536808001311

**Published:** 2008-01-16

**Authors:** B. Thimme Gowda, Miroslav Tokarčík, Jozef Kožíšek, B. P. Sowmya, Hartmut Fuess

**Affiliations:** a Department of Chemistry, Mangalore University, Mangalagangotri 574 199, Mangalore, India; b Faculty of Chemical and Food Technology, Slovak Technical University, Radlinského 9, SK-812 37 Bratislava, Slovak Republic; c Institute of Materials Science, Darmstadt University of Technology, Petersenstrasse 23, D-64287 Darmstadt, Germany

## Abstract

The conformation of the N—H bond in the structure of the title compound (N3CPBA), C_13_H_10_ClNO, is *anti* to the *meta* chloro substituent in the aniline benzene ring, similar to that observed with respect to the *ortho* chloro substituent in *N*-(2-chloro­phen­yl)benzamide (N2CPBA) and *meta* chloro substituent in *N*-(3,4-dichloro­phen­yl)benzamide (N34DCPBA), but in contrast to the *syn* conformation observed with respect to both the *ortho* and the *meta* chloro substituents in *N*-(2,3-dichloro­phen­yl)benzamide (N23DCPBA). The bond parameters in N3CPBA are similar to those in *N*-phenyl­benzamide, N2CPBA, N23DCPBA, N34DCPBA and other benzanilides. The amide group –NHCO– makes a dihedral angle of 18.2 (2)° with the benzoyl ring, while the dihedral angle between the two benzene rings is 61.0 (1)°. The mol­ecules are linked into chains along the *b* axis by N—H⋯O hydrogen bonds.

## Related literature

For related literature, see: Gowda *et al.* (2003 ▶); Gowda, Sowmya, Kožíšek *et al.* (2007 ▶); Gowda, Sowmya, Tokarčík *et al.* (2007 ▶).
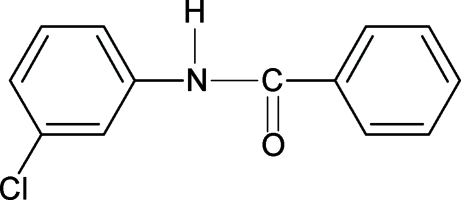


## Experimental

### 

#### Crystal data

C_13_H_10_ClNO
*M* *_r_* = 231.67
Orthorhombic, 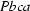

*a* = 9.3585 (2) Å
*b* = 9.7851 (2) Å
*c* = 25.1419 (6) Å
*V* = 2302.34 (9) Å^3^
*Z* = 8
Mo *K*α radiation
μ = 0.31 mm^−1^
*T* = 295 (2) K
0.41 × 0.13 × 0.06 mm

#### Data collection

Oxford Diffraction Xcalibur diffractometer
Absorption correction: analytical (*CrysAlis RED*; Oxford Diffraction, 2007 ▶). Analytical numeric absorption correction using a multifaceted crystal model (Clark & Reid, 1995 ▶). *T*_min_ = 0.915, *T*_max_ = 0.984
53566 measured reflections
2252 independent reflections
1639 reflections with *I* > 2σ(*I*)
*R*_int_ = 0.047

#### Refinement

*R*[*F* ^2^ > 2σ(*F* ^2^)] = 0.038
*wR*(*F* ^2^) = 0.101
*S* = 1.08
2252 reflections
148 parameters
1 restraint
H atoms treated by a mixture of independent and constrained refinement
Δρ_max_ = 0.19 e Å^−3^
Δρ_min_ = −0.25 e Å^−3^

### 

Data collection: *CrysAlis CCD* (Oxford Diffraction, 2007 ▶); cell refinement: *CrysAlis RED* (Oxford Diffraction, 2007 ▶); data reduction: *CrysAlis RED*; program(s) used to solve structure: *SHELXS97* (Sheldrick, 2008 ▶); program(s) used to refine structure: *SHELXL97* (Sheldrick, 2008 ▶); molecular graphics: *ORTEP-3* (Farrugia, 1997 ▶) and *DIAMOND* (Brandenburg, 2002 ▶); software used to prepare material for publication: *SHELXL97*, *PLATON* (Spek, 2003 ▶) and *WinGX* (Farrugia, 1999 ▶).

## Supplementary Material

Crystal structure: contains datablocks I, global. DOI: 10.1107/S1600536808001311/dn2311sup1.cif

Structure factors: contains datablocks I. DOI: 10.1107/S1600536808001311/dn2311Isup2.hkl

Additional supplementary materials:  crystallographic information; 3D view; checkCIF report

## Figures and Tables

**Table 1:** Hydrogen-bond geometry (Å, °)

*D*—H⋯*A*	*D*—H	H⋯*A*	*D*⋯*A*	*D*—H⋯*A*
N1—H1N⋯O1^i^	0.834 (16)	2.089 (17)	2.8989 (17)	163.5 (17)

Symmetry code: (i) 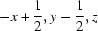.

**Fig. 1.**
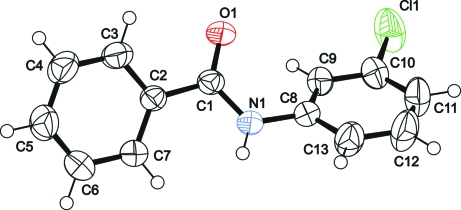
Molecular structure of the title compound showing the atom labelling scheme. Displacement ellipsoids are drawn at the 50% probability level. H atoms are represented as small spheres of arbitrary radii.

**Fig. 2.**
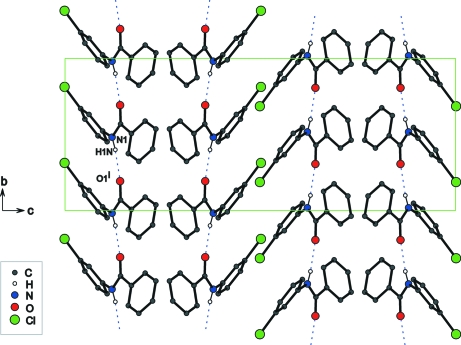
Crystal structure of the title compound viewed down the axis a. One-dimensional chains along the base vector [0 1 0] are formed by hydrogen bonds N1–H1N···O1(i). H atoms not involved in hydrogen bonding are omitted. [Symmetry code: (i) -x + 1/2, y - 1/2, z]

## supplementary crystallographic information

### Comment

In the present work, the structure of *N*-(3-chlorophenyl)-benzamide (N3CPBA) has been determined to explore the effect of substituents on the structure of *N*-aromatic amides (Gowda *et al.*, 2003; Gowda, Sowmya, Kožíšek *et al.*, 2007; Gowda, Sowmya, Tokarčík *et al.*, 2007). The conformation of the N—H bond in the structure of N3CPBA(Fig.1) is anti to the *meta* chloro substituent in the aniline phenyl ring, similar to that observed with respect to the *ortho*-chloro substituent in *N*-(2-chlorophenyl)-benzamide (N2CPBA)(Gowda, Sowmya, Kožíšek *et al.*, 2007) and *meta*-chloro substituent in *N*-(3,4-dichlorophenyl)-benzamide (N34DCPBA) (Gowda, Sowmya, Tokarčík *et al.*, 2007), but in contrast to the *syn* conformation observed with respect to both the *ortho* & *meta*-Chloro substituents in *N*-(2,3-dichlorophenyl)- benzamide (N23DCPBA)(Gowda, Sowmya, Tokarčík *et al.*, 2007). The bond parameters in N3CPBA are similar to those in *N*-(phenyl)-benzamide, N2CPBA, N23DCPBA, N34DCPBA and other benzanilides. The amide group –NHCO– has the dihedral angle of 18.2 (2)° with the benzoyl ring, while the dihedral angle between the two benzene rings (benzoyl and aniline) is 61.0 (1)°. One-dimensional chains of the title compound along the base vector [0 1 0] formed by hydrogen bonds N1–H1N···O1 (Table 1) as viewed down the *a* axis is shown in Fig.2.

### Experimental

The title compound was prepared according to the literature method (Gowda *et al.*, 2003). The purity of the compound was checked by determining its melting point. It was characterized by recording its infrared and NMR spectra. Single crystals of the title compound were obtained from an ethanolic solution and used for X-ray diffraction studies at room temperature.

### Refinement

H atoms bonded to C atoms were placed in geometrically calculated positions and subsequently treated as riding with C—H bond distance 0.93 Å. H(N) atom was visible in the difference map. In refinement the N—H distance was restrained to 0.86 (4) Å. The *U*_iso_(H) values were set at 1.2 *U*_eq_(C,*N*).

### Crystal data

**Table:**

C_13_H_10_ClNO	*F*_000_ = 960
*M**_r_* = 231.67	*D*_x_ = 1.337 Mg m^−^^3^
Orthorhombic, *P**b**c**a*	Mo *K*α radiation λ = 0.71073 Å
Hall symbol: -P 2ac 2ab	Cell parameters from 14003 reflections
*a* = 9.3585 (2) Å	θ = 3.0–29.5º
*b* = 9.7851 (2) Å	µ = 0.31 mm^−^^1^
*c* = 25.1419 (6) Å	*T* = 295 (2) K
*V* = 2302.34 (9) Å^3^	Prism, colourless
*Z* = 8	0.41 × 0.13 × 0.06 mm

### Data collection

**Table:**

Oxford Diffraction Xcalibur diffractometer	2252 independent reflections
Monochromator: graphite	1639 reflections with *I* > 2σ(*I*)
Detector resolution: 10.434 pixels mm^-1^	*R*_int_ = 0.047
*T* = 295(2) K	θ_max_ = 26.0º
φ scans, and ω scans with κ offsets	θ_min_ = 4.7º
Absorption correction: analytical(CrysAlis RED; Oxford Diffraction, 2007). Analytical numeric absorption correction using a multifaceted crystal model (Clark & Reid, 1995).	*h* = −11→11
*T*_min_ = 0.915, *T*_max_ = 0.984	*k* = −12→12
53566 measured reflections	*l* = −31→31

### Refinement

**Table:**

Refinement on *F*^2^	Secondary atom site location: difference Fourier map
Least-squares matrix: full	Hydrogen site location: inferred from neighbouring sites
*R*[*F*^2^ > 2σ(*F*^2^)] = 0.038	H atoms treated by a mixture of independent and constrained refinement
*wR*(*F*^2^) = 0.101	*w* = 1/[σ^2^(*F*_o_^2^) + (0.0491*P*)^2^ + 0.2808*P*] where *P* = (*F*_o_^2^ + 2*F*_c_^2^)/3
*S* = 1.08	(Δ/σ)_max_ < 0.001
2252 reflections	Δρ_max_ = 0.19 e Å^−^^3^
148 parameters	Δρ_min_ = −0.25 e Å^−^^3^
1 restraint	Extinction correction: none
Primary atom site location: structure-invariant direct methods	

### Special details

**Table:**

Geometry. All e.s.d.'s (except the e.s.d. in the dihedral angle between two l.s. planes) are estimated using the full covariance matrix. The cell e.s.d.'s are taken into account individually in the estimation of e.s.d.'s in distances, angles and torsion angles; correlations between e.s.d.'s in cell parameters are only used when they are defined by crystal symmetry. An approximate (isotropic) treatment of cell e.s.d.'s is used for estimating e.s.d.'s involving l.s. planes.
Refinement. Refinement of *F*^2^ against ALL reflections. The weighted *R*-factor *wR* and goodness of fit *S* are based on *F*^2^, conventional *R*-factors *R* are based on *F*, with *F* set to zero for negative *F*^2^. The threshold expression of *F*^2^ > σ(*F*^2^) is used only for calculating *R*-factors(gt) *etc*. and is not relevant to the choice of reflections for refinement. *R*-factors based on *F*^2^ are statistically about twice as large as those based on *F*, and *R*- factors based on ALL data will be even larger.

### Fractional atomic coordinates and isotropic or equivalent isotropic displacement parameters (Å^2^)

**Table:**

	*x*	*y*	*z*	*U*_iso_*/*U*_eq_	
N1	0.27214 (14)	0.48267 (13)	0.12047 (5)	0.0462 (4)	
H1N	0.2650 (19)	0.4004 (17)	0.1287 (6)	0.055*	
O1	0.19225 (12)	0.69327 (10)	0.14128 (5)	0.0565 (3)	
Cl1	0.54083 (6)	0.81350 (6)	−0.00120 (2)	0.0875 (2)	
C1	0.17675 (16)	0.56906 (15)	0.14234 (6)	0.0405 (4)	
C2	0.05098 (15)	0.50735 (14)	0.16988 (6)	0.0392 (4)	
C3	−0.02405 (19)	0.58822 (17)	0.20536 (7)	0.0546 (5)	
H3	0.0038	0.6784	0.2108	0.065*	
C4	−0.1391 (2)	0.53666 (19)	0.23252 (8)	0.0671 (5)	
H4	−0.1877	0.5916	0.2567	0.081*	
C5	−0.1830 (2)	0.4050 (2)	0.22432 (8)	0.0684 (6)	
H5	−0.2613	0.3706	0.2427	0.082*	
C6	−0.1111 (2)	0.32411 (18)	0.18890 (8)	0.0654 (5)	
H6	−0.1414	0.2349	0.1829	0.079*	
C7	0.00613 (18)	0.37454 (17)	0.16203 (7)	0.0519 (4)	
H7	0.0553	0.3185	0.1384	0.062*	
C8	0.40219 (17)	0.52388 (15)	0.09631 (6)	0.0444 (4)	
C9	0.40661 (17)	0.63258 (16)	0.06157 (6)	0.0471 (4)	
H9	0.3235	0.6796	0.0528	0.056*	
C10	0.53533 (19)	0.67062 (18)	0.04005 (7)	0.0544 (5)	
C11	0.6590 (2)	0.6017 (2)	0.05099 (8)	0.0719 (6)	
H11	0.7453	0.6288	0.0359	0.086*	
C12	0.6527 (2)	0.4918 (2)	0.08464 (10)	0.0808 (6)	
H12	0.7355	0.4428	0.092	0.097*	
C13	0.5255 (2)	0.4528 (2)	0.10771 (8)	0.0657 (5)	
H13	0.523	0.3788	0.1309	0.079*	

### Atomic displacement parameters (Å^2^)

**Table:**

	*U*^11^	*U*^22^	*U*^33^	*U*^12^	*U*^13^	*U*^23^
N1	0.0440 (8)	0.0309 (6)	0.0636 (9)	0.0007 (6)	0.0069 (7)	0.0025 (6)
O1	0.0570 (7)	0.0299 (6)	0.0827 (8)	−0.0007 (5)	0.0119 (6)	0.0004 (5)
Cl1	0.0668 (4)	0.0990 (5)	0.0968 (4)	−0.0134 (3)	0.0102 (3)	0.0423 (3)
C1	0.0405 (9)	0.0349 (8)	0.0462 (9)	0.0023 (7)	−0.0054 (7)	0.0006 (7)
C2	0.0387 (8)	0.0353 (8)	0.0437 (8)	0.0030 (6)	−0.0031 (7)	0.0027 (6)
C3	0.0621 (11)	0.0407 (9)	0.0609 (10)	0.0035 (8)	0.0091 (9)	−0.0026 (8)
C4	0.0711 (13)	0.0596 (11)	0.0707 (12)	0.0091 (10)	0.0291 (11)	0.0013 (9)
C5	0.0605 (12)	0.0658 (12)	0.0789 (13)	−0.0021 (10)	0.0232 (11)	0.0136 (10)
C6	0.0615 (12)	0.0485 (10)	0.0862 (14)	−0.0122 (9)	0.0123 (11)	0.0001 (9)
C7	0.0495 (10)	0.0433 (9)	0.0628 (11)	−0.0030 (8)	0.0084 (8)	−0.0081 (8)
C8	0.0418 (9)	0.0393 (8)	0.0521 (9)	0.0005 (7)	0.0040 (7)	−0.0044 (7)
C9	0.0392 (9)	0.0500 (9)	0.0520 (9)	−0.0007 (8)	−0.0016 (8)	−0.0003 (8)
C10	0.0489 (11)	0.0621 (11)	0.0521 (10)	−0.0090 (8)	0.0025 (8)	0.0038 (8)
C11	0.0435 (11)	0.0900 (14)	0.0824 (14)	−0.0024 (10)	0.0133 (10)	0.0107 (12)
C12	0.0461 (12)	0.0917 (15)	0.1046 (16)	0.0207 (11)	0.0101 (11)	0.0216 (13)
C13	0.0528 (12)	0.0615 (11)	0.0827 (13)	0.0112 (9)	0.0081 (10)	0.0170 (10)

### Geometric parameters (Å, °)

**Table:**

N1—C1	1.3468 (19)	C6—C7	1.380 (2)
N1—C8	1.419 (2)	C6—H6	0.93
N1—H1N	0.834 (16)	C7—H7	0.93
O1—C1	1.2244 (17)	C8—C9	1.377 (2)
Cl1—C10	1.7415 (18)	C8—C13	1.378 (2)
C1—C2	1.493 (2)	C9—C10	1.372 (2)
C2—C7	1.380 (2)	C9—H9	0.93
C2—C3	1.384 (2)	C10—C11	1.368 (3)
C3—C4	1.371 (2)	C11—C12	1.369 (3)
C3—H3	0.93	C11—H11	0.93
C4—C5	1.368 (3)	C12—C13	1.378 (3)
C4—H4	0.93	C12—H12	0.93
C5—C6	1.368 (3)	C13—H13	0.93
C5—H5	0.93		
			
C1—N1—C8	124.41 (13)	C2—C7—C6	120.57 (16)
C1—N1—H1N	116.9 (12)	C2—C7—H7	119.7
C8—N1—H1N	116.7 (12)	C6—C7—H7	119.7
O1—C1—N1	122.38 (14)	C9—C8—C13	119.78 (15)
O1—C1—C2	120.33 (14)	C9—C8—N1	121.12 (14)
N1—C1—C2	117.26 (13)	C13—C8—N1	119.10 (14)
C7—C2—C3	118.46 (15)	C10—C9—C8	119.09 (15)
C7—C2—C1	123.66 (14)	C10—C9—H9	120.5
C3—C2—C1	117.88 (13)	C8—C9—H9	120.5
C4—C3—C2	120.60 (16)	C11—C10—C9	122.00 (17)
C4—C3—H3	119.7	C11—C10—Cl1	119.38 (14)
C2—C3—H3	119.7	C9—C10—Cl1	118.60 (14)
C5—C4—C3	120.47 (17)	C10—C11—C12	118.36 (18)
C5—C4—H4	119.8	C10—C11—H11	120.8
C3—C4—H4	119.8	C12—C11—H11	120.8
C4—C5—C6	119.71 (17)	C11—C12—C13	120.98 (19)
C4—C5—H5	120.1	C11—C12—H12	119.5
C6—C5—H5	120.1	C13—C12—H12	119.5
C5—C6—C7	120.18 (17)	C8—C13—C12	119.76 (18)
C5—C6—H6	119.9	C8—C13—H13	120.1
C7—C6—H6	119.9	C12—C13—H13	120.1
			
C8—N1—C1—O1	3.0 (2)	C5—C6—C7—C2	−1.0 (3)
C8—N1—C1—C2	−175.25 (14)	C1—N1—C8—C9	−45.2 (2)
O1—C1—C2—C7	163.26 (16)	C1—N1—C8—C13	135.30 (17)
N1—C1—C2—C7	−18.5 (2)	C13—C8—C9—C10	−1.9 (2)
O1—C1—C2—C3	−17.0 (2)	N1—C8—C9—C10	178.61 (14)
N1—C1—C2—C3	161.30 (14)	C8—C9—C10—C11	1.7 (3)
C7—C2—C3—C4	0.8 (3)	C8—C9—C10—Cl1	−176.82 (12)
C1—C2—C3—C4	−178.95 (15)	C9—C10—C11—C12	−0.2 (3)
C2—C3—C4—C5	−1.1 (3)	Cl1—C10—C11—C12	178.29 (17)
C3—C4—C5—C6	0.3 (3)	C10—C11—C12—C13	−1.1 (3)
C4—C5—C6—C7	0.8 (3)	C9—C8—C13—C12	0.6 (3)
C3—C2—C7—C6	0.2 (3)	N1—C8—C13—C12	−179.87 (18)
C1—C2—C7—C6	179.94 (16)	C11—C12—C13—C8	0.9 (3)

### Hydrogen-bond geometry (Å, °)

**Table:**

*D*—H···*A*	*D*—H	H···*A*	*D*···*A*	*D*—H···*A*
N1—H1N···O1^i^	0.834 (16)	2.089 (17)	2.8989 (17)	163.5 (17)

Symmetry codes: (i) −*x*+1/2, *y*−1/2, *z*.
